# Effect of *Arrabidaea chica* extract against chemically induced breast cancer in animal model^1^

**DOI:** 10.1590/s0102-865020190100000001

**Published:** 2019-12-09

**Authors:** Keyla Borges Ferreira Rocha, Cláudia Nunes Oliveira, Ítalo Medeiros Azevedo, Robson de Macedo, Aldo Cunha Medeiros

**Affiliations:** I Fellow PhD degree, Postgraduate Program in Health Sciences, Universidade Federal do Rio Grande do Norte (UFRN), Natal-RN, Brazil. Technical procedures, acquisition of data, statistics analysis, critical revision.; II PhD, Full Professor, Nucleus of Experimental Surgery, UFRN, Natal-RN, Brazil. Conception, design, intellectual and scientific content of the study, critical revision, final approval.

**Keywords:** Breast Neoplasms, Vincristine, Models, Animal, Rats

## Abstract

**Purpose:**

To examine the effects of *Arrabidaa chica* (*Bignoniacea*) extract, a native plant of the Amazon known as crajiru, on a 7,12-dimethyl-1,2-benzanthracene (DMBA)-induced breast cancer model in Wistar rats.

**Methods:**

We compared the response of breast cancer to the oral administration of *A. chica* extract (ACE) for 16 weeks, associated or not with vincristine. Groups: normal control; DMBA (50mg/kg v.o,) without treatment; DMBA+ACE (300 mg/kg); DMBA+vincristine. 500μg/kg injected i.p; DMBA+ACE+Vincristine 250μg/kg i.p. Imaging by microPET and fluorescence, biochemistry, oxidative stress, hematology and histopathology were used to validate the treatments.

**Results:**

All animals survived. A gradual weight gain in all groups was observed, with no significant difference (p>0.05). The oral administration of ACE and ACE+vincristine 50% significantly reduced breast tumors incidence examined with PET-18FDG and fluorescence (p<0.001). Significant reduction of serum transaminases, oxidative stress and hematological toxicity were observed in these groups. Antioxidant enzyme levels in breast tissue were significantly higher compared to the DMBA and DMBA+vincristine groups.

**Conclusion:**

These results demonstrate for the first time that ACE positively influences the treatment of DMBA-induced breast cancer in animal model, inducing a reduction in oxidative stress and chemotherapy toxicity, meaning that ACE may have clinical implication in further studies.

## Introduction

Breast cancer is a major cause of morbidity and mortality among women. Worldwide, it is the second most common type of cancer. There is a tendency for increased mortality from breast cancer in Brazilian women^1^. Breast cancer originates from breast tissue, most commonly from the inner lining of milk ducts or lobes that supply the ducts, and the main metastasis pathway is the lymphatic system or the bloodstream^2^. Breast cancer may be induced by 7,12-dimethyl-1,2-benzanthracene (DMBA), a procarcinogen with selectivity for female breast cancer. It undergoes metabolic activation to carcinogenic dihydrodiolepoxide. Dihydrodiolepoxide binds to adenine residues of deoxyribonucleic acid, resulting in mutagenesis and carcinogenesis^3^.

Vincristine is a cell cycle specific anticancer drug. The cytotoxic activity of vincristine is related to inhibition of microtubules and changing of tubulin polymerization balance, which causes the cell division to stop in tumors^4^. It is part of chemotherapy protocols for breast cancer^5^.

*Arrabidaea chica* is a plant from the *Bignoniaceae* family, a native species from the Amazon region (known as crajiru) and currently occurs in the tropical regions of South America and Africa^6^. Several flavonoids and anthocyanidins have been isolated from its leaves^7^, as well as the major classes of secondary metabolites such as anthocyanins, anthraquinone, catechins, organic acids, reducing sugars, steroids, xanthones, tannins, flavanonols and flavanones^8^ ^,^ ^9^. The content of phenolic and flavonoid compounds in the leaf were determined^9^.

*A. chica* leaves have traditionally been used by Brazilian Indians as a red dye in ritual body painting, as well as anti-inflammatory, anti-anemic, analgesic and healing agent. Ribeiro *et al*.^10^ studied in mice the effect of *A. chica* extract on the evolution of Ehrlich tumors, and demonstrated significant reduction of lesions without adverse effects.

Positron emission tomography (PET) is a high-resolution imaging exam that has been very useful for cancer diagnosis and treatment follow-up^11^ ^,^ ^12^. PET is a molecular imaging technique that uses radionuclide tracer positron emitters such as 18F-fluorodeoxyglucose (18F-FDG), which allow for noninvasive assessment of metabolic and physiological activities in healthy and diseased states at molecular and cellular levels^13^. This tracer can detect glycolytic activity that is high in cancer, inflammation and infection^13^. In the present study we used *in vivo* microPET equipment for the evaluation of mammary carcinoma response to *A. chica* lyophilized extract, compared to conventional chemotherapy with vincristine. In vivo fluorescence imaging, oxidative stress, biochemical parameters and histopathology contributed to the evaluation of neoplasms.

Based on data described above, the present study aimed to investigate whether *A. chica* extract (ACE) can attenuate the development of breast carcinoma and evaluate its antitumor effect when associated with half the dose of vincristine against induced-breast carcinoma by DMBA in rats.

## Methods

The project was approved by the institutional Commission of Ethics in the Use of Animals (protocol 04/2018). All experimental procedures were performed based on the guidelines of the Brazilian College of Animal Experimentation, as well as the Brazilian Law No. 11.794/08. Wistar female rats (*Rattus norvegicus*) weighing 185±23g from the Animal Science Center, UFRN, were used. Young (six week) rats, more sensitive to DMBA, were used for breast tumor induction^14^. The rats were acclimatized for 1 week prior to the start of the experiment under standard housing conditions, including room temperature 22-24°C, relative humidity 40% and 12-hour light-dark cycle in polypropylene cages (maximum 2 animals/cage). The animals had *ad libitum* access to the rodent diet (Prevence^®^) and water.

### Extract preparation

The ethanol extraction was previously described^15^. Briefly, it was prepared by maceration of dry *A. chica* leaves (200 g). Ethanol in a 1:3 ratio was added for the percolation process at room temperature. The material was filtered and concentrated in rotary evaporator RV3 (IKA-Brazil, Campinas, SP, Brazil) at 60°C temperature. The extract was weighed and a hydroalcoholic extract at a concentration of 10% was obtained. This final extract was dried and lyophilized.

### Breast cancer induction

Induction of breast cancer was done by 7,12-dimethyl-1,2-benzanthracene (DMBA) injection (Sigma, St. Louis, MO, US) dissolved in corn oil. Except for the normal control group, all the other animals were treated with an oral single dose (50mg/kg) of DMBA. Palpation of breast tumors (twice a week) began 4 weeks after initiation of DMBA treatment. The experiment was terminated 16 weeks after DMBA administration.

### Experimental design

Thirty Wistar rats were divided into 5 groups with 6 animals each. All rats submitted to breast cancer induction received a single dose of DMBA 50 mg/kg dissolved in corn oil (1 mL) orally (v.o.) by gavage, and were observed for 16 weeks.

Group 1: Normal Control. Saline-treated rats (1 mL) v.o. for 16 weeks.

Group 2: DMBA treated rats.

Group 3: DMBA + *A. chica* extract (DMBA + ACE). Treatment with 300 mg/Kg ACE v.o. by gavage three times a week (Monday, Wednesday and Friday), until completing 16 weeks.

Group 4: DMBA + Vincristine (DMBA + VIN). VIN was injected intraperitoneally (i.p.) at a dose of 500 µg / Kg each week for 5 consecutive weeks.

Group 5: DMBA + ACE + VIN50% group. VIN 250 µg/kg was injected i.p. each week for 5 consecutive weeks + ACE 300 mg/kg by gavage three times a week (Monday, Wednesday and Friday), until completing 16 weeks.

### Weighing and survival

The animals were weighed weekly throughout the experiment period, and their survival time in days was recorded.

### 18-FDG-PETscan imaging

2-[18F]fluoro-2-deoxy-D-glucose (18F-FDG) was used. After the observation period (16 weeks), the animals were anesthetized with ketamine 70 mg/kg and xylazine 7 mg/kg i.p. The 1.5 mCi dose of (0.5 mL) 18F-FDG was injected i.v. in each animal, and dynamic imaging began 30 minutes after injection. PET three-dimensional images were captured from the whole body of the animals with the Albira microPET Preclinical Imaging System, (Bruker BioSpin Co., The Woodlands, TX, USA).

The analysis of the radiotracer uptake and respective images was performed using the equipment software (PMOD), considering the tissues and regions of interest (ROI) of the areas under study.

### Fluorescence imaging (in vivo)

Then, the anesthetized animals were injected i.v. with 0.16 mg of green indocyanine (Ophtalmos, Sao Paulo-SP, Brazil). After 24 hours, *in vivo* fluorescence images were obtained using the *in vivo*-Kodak FX Image Station, New Haven, CT, USA. The emission and excitation filters were 700 and 540nm respectively. The imaging protocol (60 second exposure time, 4x binning, f-stop 2.5, 160 mm field of view and 9 mm focal plane) was maintained for all examinations. The images were analyzed by Kodak Molecular Imaging software (version 5.0) and quantified according to color scale, as previously described^16^. Briefly, an automated tool-created region of interest (ROI) was determined around the rat's abdomen and breast areas. Signal intensities of the ROIs were expressed as arbitrary units of fluorescent signal intensity. Grayscale images were colored for representational purposes according to a color scale set for the highest and lowest levels of mean fluorescence intensity (red and purple indicate maximum and minimum light intensity, respectively).

### Biochemical analysis

Blood was collected by cardiac puncture (5 mL) of the still anesthetized animals, immediately after the imaging exams, for blood count and serum dosages. Aspartate aminotransferase (AST), alanine aminotransferase (ALT), Gamma glutamyl transferase (GGT) and albumin were dosed on a self-analyzer (Konelab, Software Version, 60i, Finland). Hematological analysis was performed using CELL-DYN Analyser (Abbott Park, Illinois, USA).

### Examination of antioxidant and oxidative enzymatic parameters

Parts of mammary tissues were homogenized in phosphate buffer solution and homogenate supernatants were used to examine the enzymatic antioxidant catalase (CAT), superoxide dismutase (SOD), glutathione peroxidase (GPx) and oxidative malondialdehyde (MDA) parameters using colorimetric assay kits, according to the manufacturer's instructions. (ABCAM, Cambridge, MA, USA).

### Histopathological exam

Euthanasia of the animals was performed with thiopental 100 mg/kg, i.p. Fresh tumor tissue samples were collected, sectioned into 5 mm thick fragments and washed in running water, allowing the fast and uniform action of the fixative solution. The specimens were fixed in 10% buffered formaldehyde for a maximum of 48 hours and then processed for 18 hours in automated tissue processor using Leica TP 1020 equipment (Leica Biosystems, BuffaloGrove, IL, USA). Histological sections were obtained with Leica microtome. RM 2125 RTS (Leica Biosystems, BuffaloGrove, IL, USA), 04 microns thick, on previously silanized slides. Tissue sections were stained with hematoxylin-eosin for morphological analysis under light microscopy (Olympus Microscope CX41, Tokyo, Japan).). The development of breast carcinoma was characterized by the presence of neoplastic cells and everything was studied by an independent pathologist, blinded to the study groups.

### Statistical analysis

The assumption of normality was assessed by the Shapiro Wilk and Kolmogorov-Smirnov test. To test the hypothesis of difference between groups, we used the Analysis of Variance (ANOVA) and the Kruskal Wallys test, followed by Tukey and Dunett multiple comparison tests, with a significance level of 5%. The statistical package SPSS^®^20 (Chicago, Ill, USA) was used.

## Results

**Figure 1:**
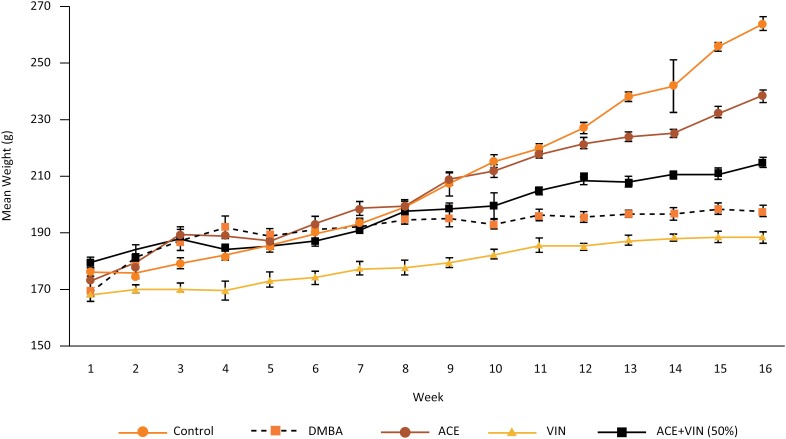
Mean weight of animals per week, by group. DMBA, dimethyl-benzanthracene; ACE, *A. chica* extract, VIN, vincristine.

All animals survived after the experimental model procedures. The evolution of body weights of the five study groups is shown in Figure 1. There was a gradual weight gain in all groups over 16 weeks. The higher weight gain was observed in the control group rats, with no significant difference between groups (p>0.05).

### PET scan imaging

**Figure 2:**
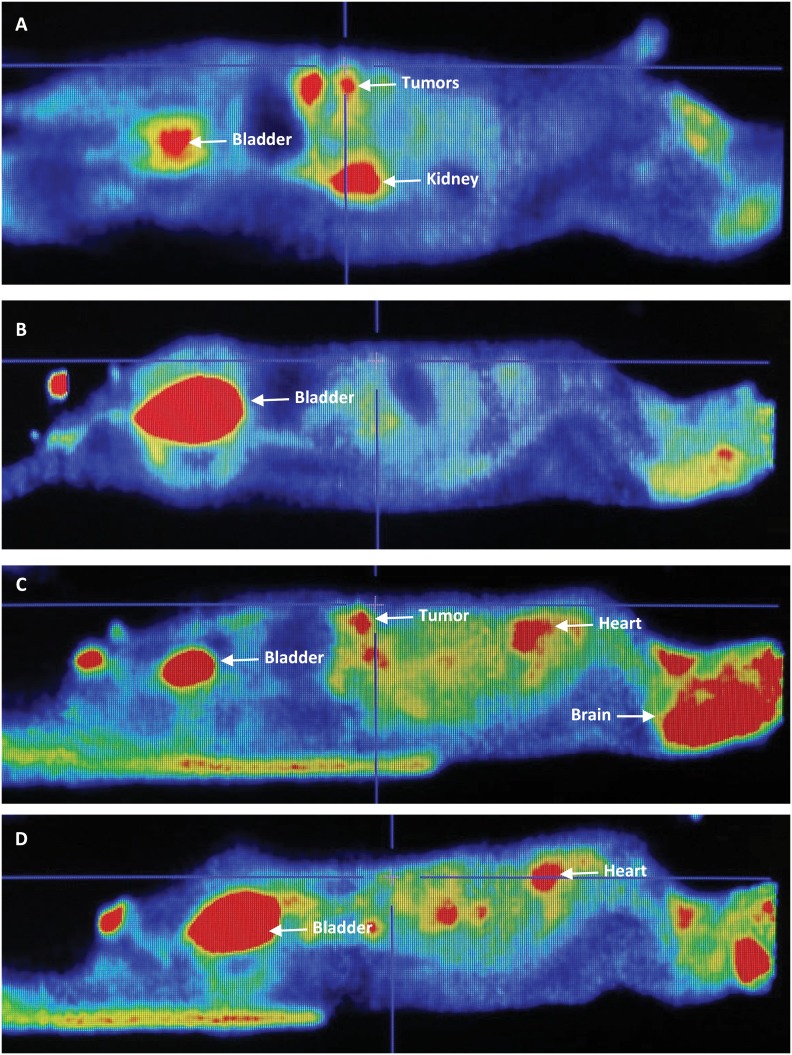
PetScan Images. Sagittal sections representative of study group rats. **A**. Shows two rat mammary tumors of the DMBA group (arrows); **B**. (DMBA + ACE group rat) no 18-FDG uptake image on the anterior abdominal wall; absence of breast tumor; **C**. (DMBA + VIN group rat) shows one breast tumor image (arrow); **D**. (rat group DMBA + ACE + VIN50%), no image with metabolic activity indicative of breast carcinoma. Other 18-FDG images represent brain, heart, kidneys and bladder (*arrows*).

Figure 2 shows images of sagittal sections taken from microPET, representative of rats from the study groups. Figure 2A shows two breast tumor nodules from the DMBA group, and Figure 2B (DMBA+ACE group) shows no image of 18-FDG uptake in the anterior abdominal wall, indicating no breast tumor. The standardized uptake values (SUV) of these images confirmed the rare presence of breast cancer, as a result of significant reduction in tumor uptake of 18-FDG and respective metabolic activity, as clearly visualized by microPET. These images coincided with the intergroup comparison of *ex-vivo* fluorescence imaging. Figure 2C (DMBA+VIN group rat) shows an image of a breast tumor and Figure 2D (DMBA + ACE + VIN50% group rat) shows no images with metabolic activity, indicative of breast carcinoma. The other images resulting from 18-FDG uptake coincide with the presence of brain, kidneys and bladder (see arrows).

### Fluorescence imaging

**Figure 3:**
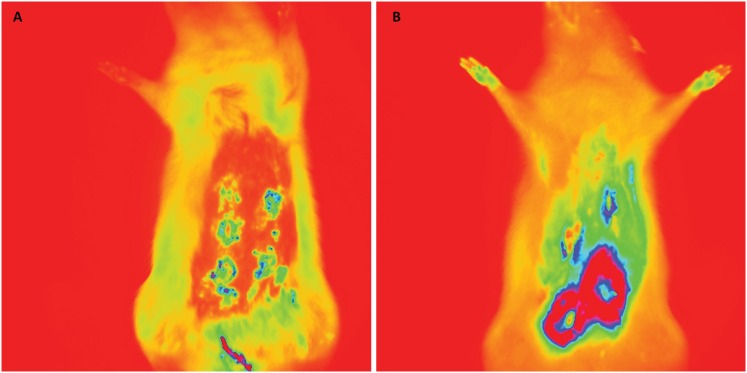
Fluorescence images. DMBA group; fluorescence after green indocyanine injection. **A**, five breast tumor images. **B**, four breast tumor images.

**Figure 4:**
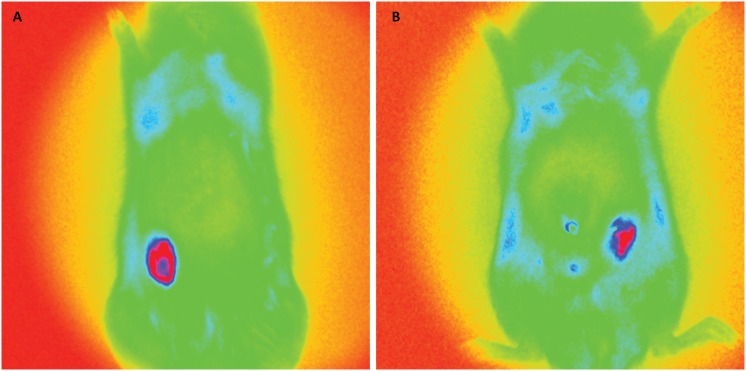
Fluorescence images (DMBA + ACE group). **A** and **B** one breast tumor in each representative image.

**Figure 5:**
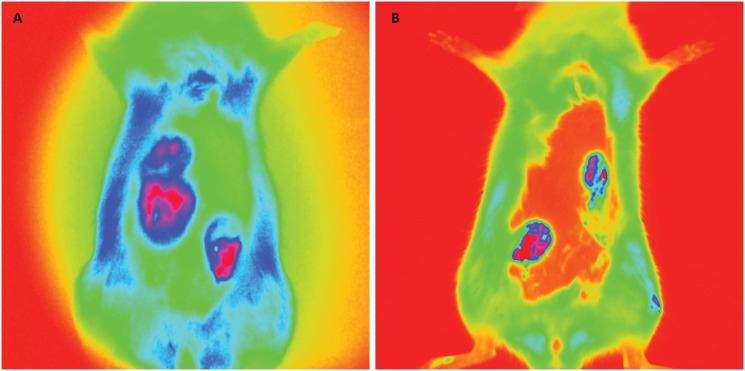
Fluorescence images (DMBA + VIN group). **A**, 3 breast tumors. **B**, 2 breast tumors.

**Figure 6:**
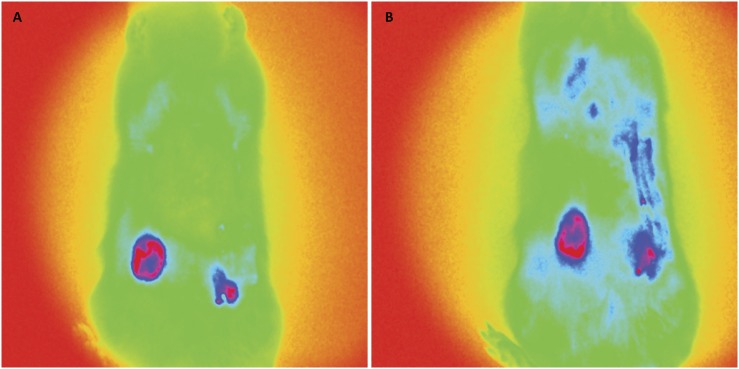
Fluorescence images (DMBA + ACE + VIN 50% group). **A** and **B**, 2 breast tumors in rats treated with ACE + VIM at half dose.

Figures 3 to 6 show *in vivo* representative images of the fluorescence intensity of induced breast tumors submitted to various treatments. In Figure 3, representative of two animals from the DMBA group (untreated), the fluorescence intensity highlighted in red indicates carcinoma, and was significantly higher than in Figures 4 to 6. The fluorescence intensity and respective areas of tumor involvement is higher in Figure 5 (from vincristine-treated mammary carcinoma), when compared to Figures 4 and 6, from rats treated with ACE and ACE+vincristine 50% respectively. Table 1 summarizes the mean fluorescence intensity values of breasts of rats submitted to induction of DMBA mammary carcinoma and respective treatments. In animals treated with DMBA+ACE the mean fluorescence intensity was significantly lower than in DMBA group and DMBA+VIN group. (p<0.001). Data are presented in logarithm of mean fluorescence intensity.

**Table 1:** Descriptive and inferential statistics of mean fluorescence intensity.

	Groups					p-value^1^
	CONTROL	DMBA	DMBA+ACE	DMBA+VIN	DMBA+ACE + VIN 50%	
MIF (log)	0.0	4.4 ± 0.55^a,b^	2.63 ± 0.59^a^	3.9 ± 0.66^a^	3.42 ± 0.3^b^	<0.001

Mean ± standard deviation.

1 p-value Kruskal Wallys.

2 Values - in the same row - followed by at least one equal letter showed statistically significant differences. (p<0.001). MIF (log), Mean intensity fluorescence (log); DMBA, dimethylbenzoanthracene; VIN, vincristine; ACE, *A. chica* extract.

### Biochemical and hematological analysis

At the end of the observation period, biochemical determinations showed significantly higher serum ALT, AST and GGT levels in animals treated with VIN, compared with rats in the ACE-treated group (p<0.001). The highest levels of ALT, AST and GGT were observed in animals after induction of DMBA mammary carcinoma without other treatments. The lowest albuminemia was observed in the DMBA and DMBA+VIN groups, levels significantly lower than in the DMBA+ACE and DMBA+VIN 50% groups (p<0.001) (Table 2).

**Table 2:** Descriptive and inferential statistics of biochemical analysis.

Parameters	Groups					p-value^1^
	Control	DMBA	DMBA+ACE	DMBA+VIN	DMBA+ACE + VIN 50%	
ALT (UI/L)	54.9 ± 3.5^a^	132.4 ± 4.2^ab^	61.0 ± 3.2^bc^	95.3 ± 4.7^ac^	60.6 ± 2.0^a^	<0.001
AST (UI/L)	62.3 ± 3.1^a^	113.4 ± 8.1^abc^	59.6 ± 2.9^bd^	87.5 ± 4.9^acd^	70.2 ± 3.1^c^	<0.001
GGT (UI/L)	8.0 ± 0.5^a^	36.9 ± 2.5^abc^	9.3 ± 0.5^bd^	25.7 ± 2.7^acd^	8.7 ± 0.6^c^	<0.001
Albumin (g/dL)	4.9 ± 0.2^ab^	2.5 ± 0.3^ac^	4.3 ± 0.4^cd^	2.6 ± 0.3^bd^	4.0 ± 0.1^ab^	<0.001

Mean ± standard deviation.

1 p-value ANOVA.

2 Values - in the same row followed by at least one equal letter showed statistically significant differences. (p<0.001). DMBA, dimethyl-benzanthracene; VIN, vincristine; ACE, *A. chica* extract; ALT, alanine-aminotransferase; AST, aspartate-aminotransferase; GGT, Gammagluthamyl transferase.

Hematological parameters showed that the rats treated with vincristine (DMBA+VIN) had significantly lower RBC, total leukocyte and neutrophil counts than in the DMBA+ACE and control groups (p<0.001). The difference between DMBA+ACE and DMBA+ACE+VIN 50% group rats was statistically insignificant (p>0.001). These data mean that the treatment with ACE changed the hematologic pattern to approximately the same as the rats in the normal-control group (Table 3).

**Table 3:** Descriptive and inferential statistics of hematological analysis.

	Groups					p-value^1^
	Control	DMBA	DMBA+ ACE	DMBA+VIN	DMBA+ACE + VIN 50%	
RBC (10^6^/mm^3^)	5.0±0.2^ab^	3.4 ± 0.3^acd^	4.4±0.3^ce^	3.2±0.38^bef^	4.6±0.3^df^	<0.001
Leukocytes (10^3^/mm^3^)	8.6±0.5^ab^	4.3±0.2^ab^	5.6±0.4^a^	3.2±0.27^ab^	5.72±0.3^b^	<0.001
Neutrofphys (10^3^/mm^3^)	4.8±0.2^a^	3.5±0.3^abc^	4.5±0.2^b^	2.5±0.13^abd^	4.7±0.2^cd^	<0.001

Mean ± standard deviation.

1 p-value ANOVA.

2 Values - in the same row followed by at least one equal letter showed statistically significant differences. (p<0.001). DMBA, dimethylbenzoanthracene; VIN, vincristine; ACE, *A. chica* extract; RBC, red blood cells.

### Oxidative stress

DMBA acted as an oxidizing agent in the DMBA-treated group and resulted in a significant reduction in the intracellular mammary tissue levels of the antioxidant catalase, SOD and GSH-px enzymes when compared to the normal control group (p<0.001). When ACE was added to animals treated with DMBA and DMBA+ACE+VIN50%, the tissue levels of the antioxidant enzymes catalase, SOD and GSH-px were restored, and these levels were significantly higher than in the DMBA group (p<0.001) (Table 4).

**Table 4:** Results of antioxidant enzyme dosage and respective inferential statistics.

	Groups					p-value^1^
	Control	DMBA	DMBA+ACE	DMBA+VIN	DMBA+ACE+ VIN 50%	
Catalase (mmol/mg)	127.9±1.6^ab^	39.5±2.2^b^	77.5±2.9^ab^	41.5±2.4^a^	68.4±2.5^ab^	<0.001
SOD (mmol/mg)	26.1±2.9^abc^	8.6±0.6^be^	19.1±1.3^ab^	11.1±0.8^ad^	20.9±1.7^cde^	<0.001
GSH-px (mmol/mg)	82±2.5^ab^	29.5±1.9^b^	59.8±2.1^ab^	29.1±1.8^a^	49.3±1.3^ab^	<0.001

Mean ± standard deviation.

1 p-value ANOVA.

2 Values - in the same row followed by at least one equal letter showed statistically significant differences. (p<0.001). DMBA, dimethylbenzoanthracene; VIN, vincristine; ACE, *A. chica* extract; SOD, Superoxide desmutase; GSH-px, Glutathione peroxidase

DMBA group rats had significantly higher levels of maloialdehyde (MDA) when compared to normal control rats. ACE treatment caused a significant decrease of MDA levels, which confirms the antioxidant property of the extract (p<0.001). DMBA+VIN group rats maintained the preventive power against lipid peroxides represented by MDA. When the ACE was added to animals treated with 50% of the vincristine dose (DMBA + ACE + VIN 50% group), the breast tissue MDA dosage was significantly lower than in the DMBA+VIN group (p<0.001) (Table 5).

**Table 5:** Determination of cellular index of lipid peroxidation in breast tissue homogenates.

	Groups					p-value^1^
	Control	DMBA	DMBA+ACE	DMBA+VIN	DMBA+ACE+VIN 50%	
MDA (nmol/mg)	18.4±1.7^ab^	85.8±2.6^ab^	25.7±3.1^a^	38.3±2.1^ab^	27.4±1.3^b^	<0.001

Mean ± standard deviation.

1 p-value ANOVA.

2 Values - in the same row followed by at least one equal letter showed statistically significant differences. (p<0.001). DMBA, dimethyl-benzanthracene; VIN, vincristine; ACE, *A. chica* extract; MDA, Malondialdehyde.

## Histopathology

**Figure 7:**
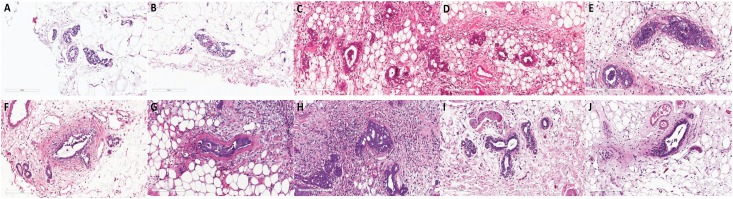
Histpathology HE, 400x magnification. **A, B**. Control group - Histologically normal breast, with a predominance of adipose tissue. Acinar and ductular structures forming mammary lobes; **C, D**. DMBA group, proliferation of atypical ducts, associated with peritumoral inflammatory response. **E, F**. DMBA + ACE group, presence of apoptosis, with mild to moderate inflammatory response. **G, H**. DMBA + VIN group, shows apoptotic cells and dense peritumor inflammation. **I, J**. DMBA + ACE + VIN50% group, tumor apoptosis with mild to moderate peritumoral inflammatory response.

Normal structure with well-defined architecture was observed in the mammary tissue of rats of the normal control group. Rats from the DMBA-induced cancer group showed edema, neutrophil inflammation, and epidermal ulceration. Ductal carcinoma with irregular cytoplasm, glandular cell multiplication and focal proliferation were found in all rats of this group. DMBA+ACE group rats showed improvement in the histological characteristics of the breast tissue. Neutrophil inflammation was observed in the vicinity of the tumor. Ductal and epidermoid carcinomas were found in ducts. Extensive apoptotic figures have been identified in the tumors. In the DMBA+full-dose vincristine group adiponecrosis was identified near the tumor areas. Apoptotic figures were present in ductal and epidermoid carcinoma. When breast tumors were treated with DMBA+ACE+VIN50%, chronic inflammation was present in the vicinity of neoplastic lesions. Apoptotic figures were identified in all ductal carcinomas (Fig. 7 A-J).

## Discussion

Breast cancer is the most common cancer among women and the second leading cause of cancer-related deaths worldwide. In 2012, 522,000 women died of breast cancer in developed and developing countries^17^.

The mechanism by which vincristine induces tumor cell death is the polymerization of mitotic microtubules and the consequent arrest of cell division in metaphase^18^. Several mechanisms have been reported to mediate vincristine-induced cytotoxicity, including its ability to induce apoptosis via oxidative stress and inflammation^19^ ^-^ ^21^. In addition, vincristine is capable of interfering with vascular blood flow and inducing cell necrosis^22^. However, vincristine has been reported to be highly toxic to other non-neoplastic tissues and cause respiratory failure, neuromuscular, cardiac and gastrointestinal toxicities^23^. Signs of hepatotoxicity, including significantly high levels of hepatic transaminase, centrilobular hemorrhagic necrosis, and histological and ultrastructural changes have been observed in patients or animal models treated with vincristine^24^. In our study vincristine was associated with oxidative stress, apoptosis and peritumoral inflammation.

The maximum penetration depth of near-infrared fluorescence in tissues is up to 1 cm, so identifying deep tumors with fluorescence imaging is difficult^16^. As in the present study, we examined superficial breast cancer lesions in rats, fluorescence images were clear and useful for the interpretation of the findings. 18F-FDG micro PET was used to monitor the therapeutic effect of ACE and vincristine in the DMBA-induced breast cancer model. The images confirmed that ACE alone or associated with half the dose of vincristine reduced the presence and volume of breast tumors. Thus, from the 18F-FDG PET results, we found that in the ACE group, and in the ACE+50% of the vincristine dose, 18F-FDG tumor uptake decreased when compared to the DMBA group.

In the present study we found that animals with DMBA-induced cancer treated with vincristine had significantly higher levels of ALT, AST and GGT than in the ACE-treated group. We observed a significant reduction in liver and hematologic toxicity in the group treated with 50% of the vincristine dose associated with ACE, with good results in the control of breast carcinoma. Treatment with ACE raised leukocyte and RBC levels. Relevant data is that in the DMBA + ACE + VIN50% group we were able to reduce the treatment toxicity by using 50% of the vincristine dose without compromising the antineoplastic effect. Low albumin levels were expected in tumor rats, which was evidenced in the DMBA and DMBA + VIN groups. In rats treated with ACE, serum albumin and transaminase levels showed significant improvement, probably due to the effect of its antioxidant components (anthocyanins, anthraquinone, catechins, organic acids, reducing sugars, steroids, xanthones, tannins, flavanonols and flavanones) in reducing oxidative stress. The antioxidant components of *A. chica* have been studied^7^ ^-^ ^9^.

In our study, we evaluated oxidative stress in DMBA-induced breast tumors in rats undergoing antitumor treatments. Our results indicated considerable oxidative stress by determining MDA in breast tumor samples taken from DMBA treated animals. Malondialdehyde (MDA) is the lipid marker of oxidative stress most commonly used to validate ischemia/reperfusion and cancer^25^. We have demonstrated that DMBA-injected rats developed oxidative stress in breast tissues, as evidenced by increased MDA levels. Oral administration of ACE to rats following DMBA-induced breast cancer reduced MDA dosage in mammary tumors. CAT, GPx and SOD are vital enzymes and antioxidant parameters^26^. In our investigation, we observed that ACE increased breast tissue CAT, GPx, and SOD enzymes in DMBA-induced breast cancer. These findings demonstrated that ACE alone or associated with vincristine can attenuate reactive oxygen species and reduce oxidative destruction of mammary tissues. These effects can be attributed to the flavonoid mixture in the plant extract^27^. Our findings suggest that ACE has a protective effect against DMBA-induced oxidative stress in breast cancer tissues. Michael *et al*.^28^ found that the ethanolic extract of *A.chica* showed in vitro antiproliferative activity against human cancer cells. We found numerous apoptotic cells in the histopathological study in the breast tissue samples of the ACE treated animals. Probably, apoptosis was the predominant mechanism of cell death in the tumors of these groups. Future studies will be needed to confirm these findings.

Studies on the antineoplastic effects of ACE, derived from herbal medicine of Amazonian origin, are scarce. However, for the first time, we proved in this study its positive effect on the control of induced breast cancer in an animal model. It does not mean that ACE can cure breast cancer. However, it was demonstrated in the present study that, if used orally alone or in combination with half the vincristine dose (VIN50%), it can contribute to the reduction of hematological toxicity and disease control, which is very relevant.

## Conclusions

ACE acted as a therapeutic agent in a rat breast cancer model. The combination of ACE and chemotherapy positively influenced the treatment of breast tumors, reduced the effective dose of chemotherapy and attenuated some of its adverse effects.
